# Effects of hypertension on maternal adaptations to pregnancy: experimental study on spontaneously hypertensive rats

**DOI:** 10.1590/S1516-31802001000200003

**Published:** 2001-03-02

**Authors:** José Carlos Peraçoli, Marilza Vieira Cunha Rudge, Maria Salete Sartori, Roberto Jorge da Silva Franco

**Keywords:** Hypertension, Pregnancy, Volume expansion, SHR strain, Hipertensão, Gestação, Expansão de volume, Linhagem SHR

## Abstract

**CONTEXT:**

Animal models for essential hypertension have been used for understanding the human pathological conditions observed in pregnant hypertensive women.

**OBJECTIVE:**

o study the possible effects of pregnancy on hypertension and of hypertension on pregnancy in spontaneously hypertensive rats (SHR), and in their normotensive Wistar-Kyoto (WKY) counterparts.

**TYPE OF STUDY:**

Comparative study using laboratory animals.

**SETTING:**

Animal Research Laboratory of Clinical Medicine at the Medical School of Botucatu, São Paulo State University, Brazil.

**SAMPLE:**

Ten to twelve-week-old virgin female normotensive Wistar-Kyoto (WKY) rats and spontaneously hypertensive rats (SHR). The animals were separated into four groups: 15 pregnant spontaneously hypertensive rats (SHR-P), 10 non-pregnant spontaneously hypertensive rats (SHR-NP), 15 pregnant normotensive rats (WKY- P), and 10 non-pregnant normotensive rats (WKY-NP).

**MAIN MEASUREMENTS:**

The blood pressure was evaluated by the tail cuff method, in rats either with or without prior training for the handling necessary for tail cuff measurements. The maternal volemia expansion was indirectly evaluated by weight gain, and by systemic parameters as hematocrit, hemoglobin, total protein, albumin and sodium retention. The perinatal outcome of pregnancy was evaluated by analysis of resorptions, litter size, rate of low weight and number of stillbirths.

**RESULTS:**

The late fall in blood pressure in the pregnant SHR strain and in the normotensive WKY strain can only be detected in rats previously trained to accept the handling necessary for the tail cuff measurement. During pregnancy the body weight gain was significantly higher in WKY than in SHR rats. Systemic parameters were significantly lower in pregnant WKY rats than in nonpregnant WKY rats, while no differences were observed between pregnant and non-pregnant SHR groups. In pregnant WKY rats the sodium retention was higher from the 13^th^ day onwards, while in SHR rats this occurred only on the 21^st^ day. The characteristics of reproductive function such as number and weight of fetus, perinatal mortality and the resorption rate were significantly affected in the SHR strain.

**CONCLUSION:**

The SHR strain may be considered as a model for chronic hypovolemic maternal hypertension, with the fetal growth retardation being determined by this hypovolemic state.

## RESUMO

**CONTEXTO:**

Os modelos animais de hipertensão arterial têm sido usados para a compreensão das condições patológicas humanas encontradas nas mulheres grávidas hipertensas.

**OBJETIVO:**

Estudar os possíveis efeitos da prenhez sobre a hipertensão arterial, e desta sobre a prenhez, em ratas espontaneamente hipertensas (SHR) e seu controle normotenso Wistar-Kyoto (WKY)

**TIPO DE ESTUDO:**

Estudo comparativo com animais de laboratório.

**LOCAL:**

Laboratório de pesquisa experimental da Faculdade de Medicina de Botucatu, Universidade Estadual de São Paulo, Brasil.

**AMOSTRA:**

Foram utilizadas ratas virgens, das linhagens WKY e SHR, com idade entre 10 e 12 semanas. Os animais foram divididos em quatro grupos: 15 SHR-prenhe (SHR- P), 10 SHR não-prenhe (SHR-NP), 15 WKY prenhe (WKY-P) e 10 WKY não-prenhe (WKY-NP).

**VARIÁVEIS ESTUDADAS:**

A pressão arterial foi avaliada pelo método do esfigmomanômetro de cauda em ratas adaptadas ou não previamente ao aquecimento necessário para a medida da pressão arterial da cauda. A expansão da volemia foi avaliada indiretamente pelo ganho de peso e por parâmetros sistêmicos como hematócrito, hemoglobina, proteínas totais, albumina e balanço de sódio. O resultado perinatal foi avaliado pelo número de nódulos de reabsorção, tamanho da ninhada, taxa de fetos com baixo peso e número de natimortos.

**RESULTADOS:**

A queda da pressão arterial no final da prenhez, das ratas SHR e WKY, ocorreu somente nas ratas adaptadas ao aquecimento para a medida da pressão arterial na cauda. Durante a prenhez o ganho de peso materno foi significativamente maior nas ratas WKY. Os parâmetros sistêmicos foram significativamente menores nas ratas WKY prenhez em relação ao seu controle não-prenhe, não sendo observada diferença significativa entre as ratas SRH prenhes e não-prenhes. Nas ratas WKY prenhez a retenção de sódio foi maior desde o décimo terceiro dia de prenhez, e nas ratas SHR apenas a partir do décimo nono dia. Os aspectos reprodutivos como número e peso dos fetos, mortalidade fetal e número de nódulos de reabsorção foram significativamente afetados na linhagem SHR.

**CONCLUSÃO:**

A linhagem SHR pode ser considerada como modelo de hipertensão arterial crônica hipovolêmico, sendo a restrição do crescimento fetal observada, conseqüente a esse estado de hipovolemia materna.

## INTRODUCTION

The understanding of abnormalities in human pathological conditions is greatly helped by the use of experimental animals and specific animal models of the disease. The spontaneously hypertensive rat (SHR) of Okamoto & Aoki,^1^ has been extensively used in hypertension research, as an experimental model for essential hypertension in humans.^2-6^

In this animal model the behavior of blood pressure during pregnancy has been a matter of controversy in the literature. Thus, no effects of pregnancy on pressure levels^7,8^ or any fall in pressure level during the last days of pregnancy have been reported.^5,6,9-11^

There is also no agreement about hemodynamic changes occurring in pregnant SHR rats, since both blood and plasma volumes have been considered to be increased, decreased or unchanged during pregnancy.^10^

It has been reported that the hypertension during pregnancy could adversely affect the fetus,^12-14^ with an inverse correlation occurring between maternal blood pressure and litter size.^5^

The aim of the present study was to follow spontaneously hypertensive rats during pregnancy and to detect the possible effects of pregnancy on hypertension and of hypertension on pregnancy.

## METHODS

### Animals

Ten to twelve-week-old virgin female normotensive Wistar-Kyoto (WKY) rats and spontaneously hypertensive rats (SHR) were purchased from Taconic Farms Inc., Germantown, NY, and then maintained in the Animal Research Laboratory for Clinical Medicine at the Medical School of Botucatu, São Paulo State University. The animals were housed four per cage in a room with temperature maintained at 22°C ± 1°C and with a 12-hour-light/12-hour- dark cycle. All the animals had free access to food and water throughout the study.

### Experimental design

Before the experiment, the animals were allowed to habituate to laboratory conditions for one week. For breeding, females were caged with males of the same strain (ratio 1:1), and vaginal smears were checked each morning for the presence of spermatozoa. The day that vaginal smears were sperm-positive was designated as day 0 of pregnancy. Age-matched, unmated females of each strain were used as non-pregnant SHR and WKY rats.

The animals were separated into four groups: 15 pregnant spontaneously hypertensive rats (SHR-P), 10 non-pregnant spontaneously hypertensive rats (SHR-NP), 15 pregnant normotensive rats (WKY-P), and 10 non-pregnant normotensive rats (WKY-NP). During the experiment the rats were housed singly in stainless steel wire-bottom cages to control food ingestion. The food had a sodium concentration of 90.2 mg/g of diet. The animals received water *ad libitum*.

### Blood pressure and body weight measurement

Systolic blood pressure was measured on days 0,7,9,11,13,15,17,19 and 21 by tailcuff plethysmography after pre-warming the rats at 37°C for 10 minutes. Each blood pressure value reported was the mean of three determinations that were taken during the same session. A level of 150 mmHg or higher was considered to be hypertension.^1^ Body weight was evaluated on days 0,7,14 and 21 of pregnancy.

Another experimental group of 10 animal each from the SHR-NP, SHR-P, WKY-NP and WKY-P groups was submitted only to measurement of systolic blood pressure on days 0, 7,14 and 21.

### Evaluation of systemic parameters

The systemic parameters were evaluated on day 21. Before sacrifice, a tail vein blood sample was obtained for the measurement of hematocrit and hemoglobin by the micromethod in a capillary tube and by the cyanomethemoglobin method, respectively. The following additional determinations were done in a blood sample obtained at sacrifice: total protein and albumin by the biuret method.^15^

### Sodium balance

The sodium retention was evaluated during the experiment over 7 periods of 3 days each, being expressed as the difference between dietary sodium ingestion and sodium excretion in urine and feces. Sodium concentration was determined by flame spectrophotometry (Carl Zeiss).

### Sacrifice and litter analyses

On day 21, before delivery, animals were anesthetized with ethyl ether and a midline laparotomy was performed to obtain the fetuses and placentas. The following parameters of pregnancy outcome were evaluated: resorptions, litter size, number of stillbirths and neonatal survival. Fetal viability was determined by response to touch or spontaneous movements. After removal of the uterus and separation from the placenta, the newborn pups were weighed and classified as small (SGA), appropriate (AGA) and large (LGA) for gestational age as compared to the mean ±SD for the control group.^16^ After the sacrifice of the fetuses, brain, liver and carcasses were collected and weighed. The placentas were separated from their membranes, weighed and fixed with formalin, paraffin embedded, cut into 4 mm sections and stained with hematoxylin-eosin.

### Statistical analysis

Data from systolic blood pressure, body weight, systemic parameters and sodium balance studied in SHR and WKY rats were analyzed by one-way variance analysis (ANOVA), followed by the Tukey method. The reproductive performance of both SHR and WKY pregnant groups were compared by Student's *t* test.^17^ The significance level was set at P < 0.05 for all tests.

## RESULTS

**Figure 1:**
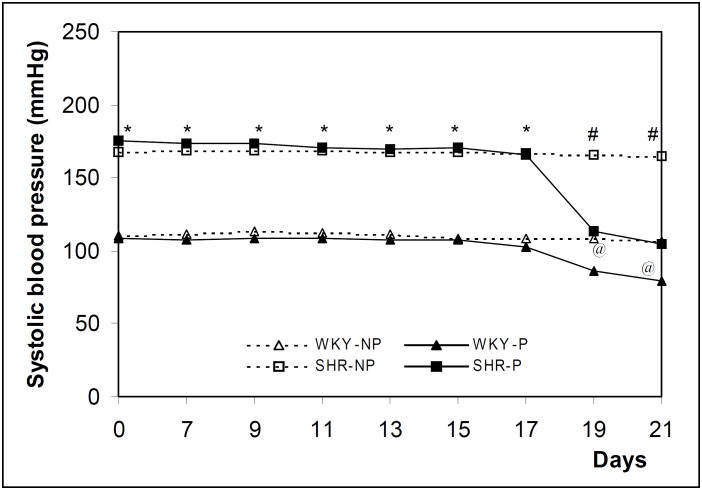
Blood pressure levels of SHR and WKY rats measured every second day of the experiment from day 7 up to day 21. The results are expressed as the mean of 12 animals evaluated per period. * P < 0.05 in relation to WKY strain; # P < 0.05 in relation to SHR-P, WKY-NP and WKY-P and @ P < 0.05 in relation to WKY-NP (ANOVA).

**Figure 2:**
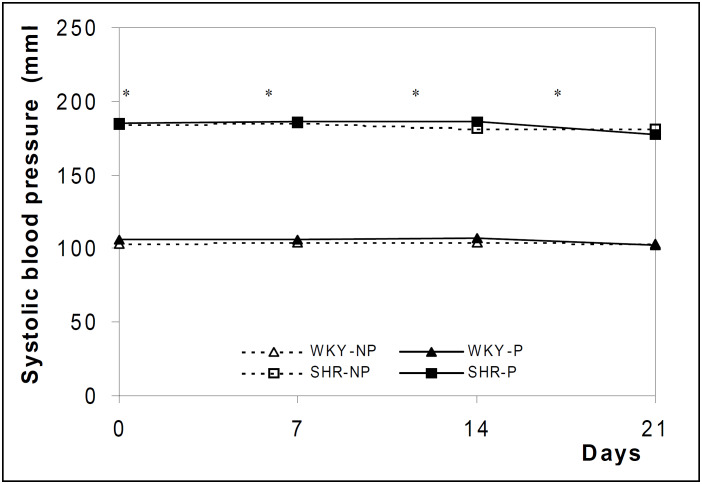
Blood pressure levels of SHR and WKY rats measured on days 0,7,14 and 21 of the experiment. The results are expressed as the mean of 10-15 animals evaluated per period. * P < 0.05 in relation to WKY strain (ANOVA)

The mean values of systolic blood pressure detected during the experiment for both SHR and control WKY rats are shown in Figures 1 and 2. The values in SHR were significantly higher than in WKY at all periods evaluated in the non-pregnant animal groups.

In pregnant rats, when systolic blood pressure was measured every other day, from day 7 onwards, a definite fall was observed in late pregnancy. Systolic blood pressure began to fall after day 17 of pregnancy in both strains studied, but the fall was more marked in the SHR strain (Figure 1). On the other hand, in the experiment in which systolic blood pressure levels were measured only on days 0,7,14 and 21 of gestation, no changes were observed in either strain studied (Figure 2).

**Figure 3:**
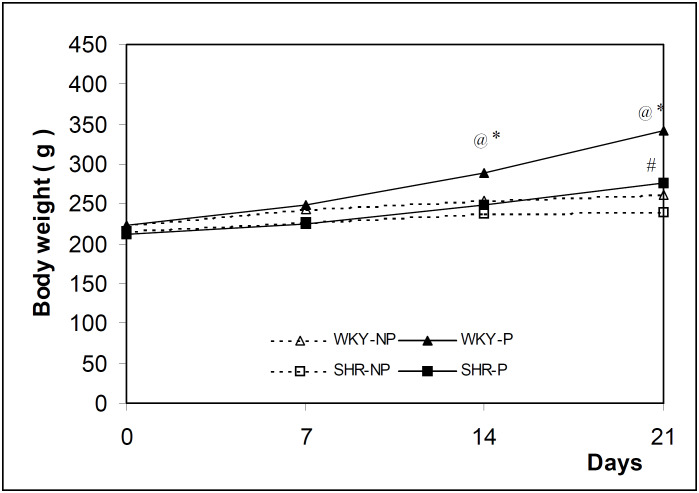
Body weight of the SHR and WKY rats evaluated on days 0, 7, 14 and 21 days of the experiment. The results are expressed as the mean of 12 animals evaluated per period. * P < 0.05 in relation to WKY-NP; # P < 0.05 in relation to SHR-NP and @ P < 0.05 in relation to SHR-P (ANOVA).

**Figure 4:**
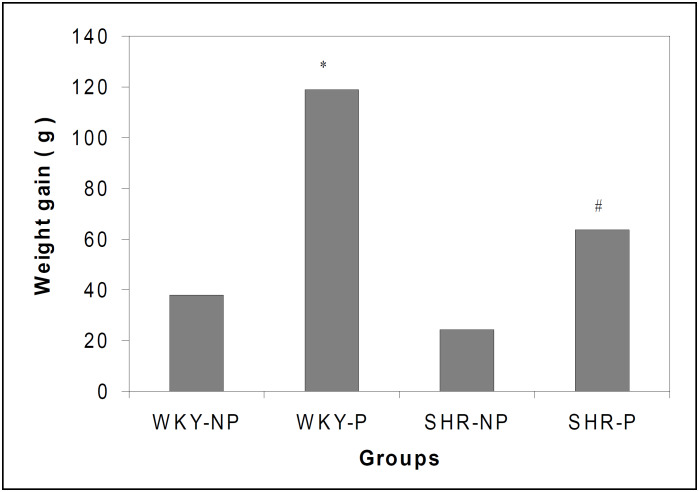
Weight gain of WKY and SHR rats measured on day 21 of the experiment. The results are expressed as mean of 10-15 animals. * P < 0.05 in relation to WKY-NP, SHR-NP and SHR-P, and # P < 0.05 in relation to SHR-NP and WKY-NP (ANOVA).

Figure 3 illustrates the body weight of the four groups of rats. Both pregnant groups presented an increase in body weight during the experiment in relation to the non-pregnant controls. The WKY-P showed significantly higher body weight from day 14 onwards, while in the SHR-P group this increase in body weight was seen only on day 21 of pregnancy. The comparison between WKY and SHR pregnant groups showed significant differences on days 14 and 21. The body weight gain obtained at the end of the experiment was higher in pregnant rats of both strains. However, in the WKY- P this weight gain was significantly higher than in SHR-P rats (Figure 4).

Table 1 summarizes the systemic parameters evaluated. The values of hematocrit, hemoglobin, total protein and albumin were significantly lower in WKY-P than in WKY-NP rats, while there were no significant differences between the pregnant and non-pregnant SHR groups.

**Table 1:** Systemic parameters determined in pregnant and control WKY and SHR rats

Systemic Parameters	Experimental groups			
	WKY-NP	WKY-P	SHR-NP	SHR-P
Hematocrit (%)	43.6	39.0*	39.7	38.8
Hemoglobin (mg%)	12.8	11.1*	12.7	11.8
Total protein (mg%)	6.2	5.1*	5.7	5.3
Albumin (mg%)	3.4	2.7*	3.3	2.9

* P < 0.05 in relation to WKY-NP (ANOVA).

**Figure 5:**
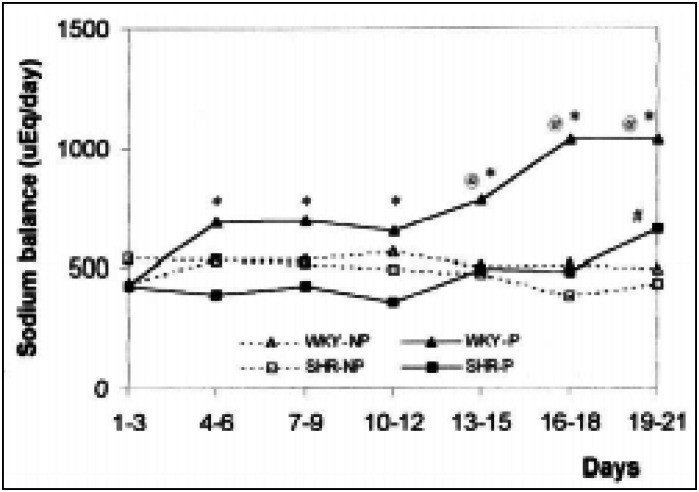
Sodium balance of the SHR and WKY rats measured in different periods of the experiment. The results are expressed as mean of 10-15 animals evaluated per period. * P<0.05 in relation to SHR-P; @ P<0.05 in relation to WKY-NP; # P<0.05 in relation to SHR-NP (ANOVA)

The effect of pregnancy on sodium retention is presented in Figure 5. WKY-P rats showed higher sodium retention than SHR-P from the 4^th^ day until the end of the study. In WKY-P the sodium retention was higher than in WKY-NP from the 13^th^ day onwards, while in SHR-P this occurred only at the last period of pregnancy.

Reproductive performance of the pregnant rats is summarized in Table 2. Litter size, fetal weight as well as the carcass, brain and liver weights of the pups from SHR rats were lower than in the WKY ones. On the other hand, the rate of resorptions, stillbirths and small for gestational age were higher in SHR pups than in the WKY group.

**Table 2:** Reproductive performance od the pregnant rats.

Reproductive Performance	Experimental groups	
	WKY	SHR
Number of fetuses	12	9*
Weight	4.92	3.61*
Carcasse weight	2.701	1.851*
Liver Weight	0.274	0.186
Resorptions	7 (4.0%)	14 (9.4%)*
Stillbirth	0	6*
SGA	25 (15.2%)	129 (100.0%)*
Placenta weight	0.414	0.397

* P < 0.05 in relation to WKY (Student).

There were no differences between SHR and WKY in relation to placenta weight (Table 2) and the histopathological studies, which showed no obvious differences between the labyrinth, junction zone and decidua basalis.

## DISCUSSION

The literature is controversial regarding the behavior of blood pressure during pregnancy in rat experimental models. Some data show no effect of pregnancy on blood pressure in the SHR strain^7-8^ or WKY strain,^10,18-20^ whereas others show a significant fall in blood pressure in the SHR strain,^5,9,10,21-23^ and/or in the WKY strain.^12,23-25^ In our study, blood pressure fell in the pregnant WKY and SHR groups, and this fall was dependent on the frequency at which blood pressure was measured, suggesting that stress can influence the behavior of blood pressure. Thus, when the rats are constantly handled they become adapted and show their real blood pressure. The SHR strain shows an exaggerated sympathetic response when submitted to stress.^26-28^ Frequent immobilization causes adaptations in the SHR rat, with a decrease in blood cathecolamines.^29^ The same might be suggested for the WKY strain. We believe that there is an advantage in the use of serial measurements at short intervals to determine the effect of pregnancy on blood pressure. This aspect might reflect what occurs in clinical practice, when normal and hypertensive pregnant women need to become familiarized with blood pressure determinations to avoid the white coat syndrome.

The explanation for the fall in blood pressure occurring in pregnant rats is unclear. A number of hypothesis have been raised, such as the number of fetuses,^5,23,30^ fetal kidney,^21^ effects of some hypotensor substances in the placenta,^21^ the angiotensin-renin system effect^23,31,32^ and endothelium-derived relaxing factor activity.^33^

Maternal volemia was indirectly evaluated by weight gain, blood measurements and sodium balance. Pregnant WKY rats increased weight from the first week of pregnancy onwards, whereas in pregnant SHR the increase in the weight was different from the non-pregnant control only at the 21^st^ day of pregnancy. The results suggest that placental and fetal weights and pregnancy adaptations were responsible for this greater weight gain in the WKY strain. In the SHR strain the inappropriate increase in maternal volemia may be attributable to the lower intensity of adaptations to pregnancy. Maternal blood measurements are an indirect way of determining pregnant volemia. Our results show that dilution of the maternal volemia in WKY rats occurred, since blood measurements showed lower values than in non-pregnant controls. On the other hand, these alterations did not occur in the SHR strain, suggesting that there was no volemia increase, or if it occurred, the increase was not detectable.

The sodium balance in WKY-P rats was higher than that observed in WKY-NP controls from the 4^th^ day of the experiment onwards. In pregnant SHR, this occurred only between days 19 and 21. This finding shows that the sodium retention, necessary for the establishment of an increase in maternal volemia occurs later in SHR rats. The sodium balance reinforced the hypothesis that volemia increased in WKY pregnant rats, occurring with high intensity and earlier than in pregnant SHR.

The aspects of reproductive function, such as number and weight of fetus, perinatal mortality and the resorption rate were significantly affected by the hypertensive state of the mothers. However, this did not occur with morphological and histological aspects of the placenta. Thus, the placenta shows normal or high growth when trying to maintain the nutrition supply of the fetus. However, this adaptation was not efficient, since the fetuses had a bad outcome.

In conclusion, the present results showed that pregnancy had a hypotensor effect on both the WKY and SHR rat strains, with this effect being greater in SHR. Blood pressure behavior was dependent on the intervals between measurements and was related to animal stress. The adaptations of volemia during pregnancy were less intense in SHR rats, which showed lower weight gain and sodium retention and no alterations in blood measurements. The SHR strain may be considered to be a model for hypovolemic chronic maternal hypertension, with fetal growth retardation being determined by this hypovolemic state.
